# Invisible body illusion modulates interpersonal space

**DOI:** 10.1038/s41598-017-01441-9

**Published:** 2017-05-02

**Authors:** Mariano D’Angelo, Giuseppe di Pellegrino, Francesca Frassinetti

**Affiliations:** 1 Department of Psychology, University of Bologna, Bologna, Italy; 2 CsrN, Centre for studies and research in Cognitive Neuroscience, University of Bologna, Bologna, Italy; 3 Istituti Clinici Scientifici Maugeri, Hospital IRCCS, Castel Goffredo, Mantova Italy; 4 School of Psychology, Bangor University, Bangor, UK

## Abstract

Interpersonal space (e.g., IPS) refers to the physical distance individuals maintain from others during social interactions, and into which intrusion by others can cause discomfort. Here, we asked whether the size of IPS is affected by manipulation of one’s own body representation. To address this issue, in Experiment 1, IPS was measured through a comfort-distance task, before and after eliciting the illusion of owning an invisible body. To rule out a general, nonspecific change in space perception consequent the illusion, we also assessed peripersonal space, e.g., PPS, the area around the body used to act on nearby objects, through a reaching-distance task. Results showed that the experience of invisibility induces a selective contraction of IPS, without affecting the perceived reaching space around the body. In Experiment 2, a tool-use manipulation produced the opposite dissociation, modifying the boundaries of PPS, but leaving IPS distance unaltered. Collectively, these findings support a close relationship between IPS and the conscious representation of the body external appearance, i.e. the body image, and suggest the existence of two functionally separate representations of the space immediately surrounding the body in humans, which may form the basis of distinct processes engaged for different behavioural contexts.

## Introduction

The term interpersonal space (IPS) refers to the protective, safety zone that people maintain around their body during social interaction, and into which intrusion by others may cause discomfort^1, 2^. The spatial extent of IPS may vary across culture^3^ and its boundaries are regulated and constantly negotiated according to the context and emotional states of individuals^4^. For instance, IPS may reduce after a cooperative social interaction^5, 6^, or after inducing a positive emotional experience^7^. Thus, studies on IPS have predominantly focused on how social space is modulated by high-order social and cognitive factors concerning the perception of the context or the attitude toward the interacting parts^8–13^.

On the other hand, a number of studies on embodied cognition have emphasized the importance of one’s own body representation in interpersonal attitudes^14–20^. Perceived bodily similarity between self and others may change the way in which subjects interact with other people^16–18^, thereby revealing the social valence of body representation^19^, and the intimate relationship between basic, body perceptual representation and the complex mechanisms underlying our everyday social interactions. For instance, Peck and co-workers^15^ demonstrated that inducing the illusion of ownership over a dark skinned virtual body reduces the implicit racial bias. In the same way, Yee and Bailenson^20^ found that participants were more willing to make unfair splits in an ultimatum game when they experienced the embodiment toward a taller than a shorter virtual body.

Recently, Guterstam *et al*.^21^, modifying the now classical rubber hand illusion, through a multisensory visuotactile conflict, created the illusion of having an invisible hand. A subsequent study from the same laboratory extended the illusion of having an invisible limb to an entire invisible body^22^. More importantly for our present purpose, authors demonstrated that the illusion of owning an invisible body, as compared to a mannequin’s body, reduced participant’s heart rate and level of subjective stress in response to standing in front of an audience of strangers. Therefore, authors concluded that this body illusion has unique effect on social-affective cognition. Indeed, being gazed upon constitutes a salient social cue, and perceiving one’s own body as invisible can affect socio-affective processing of such cues^22^.

Based on these findings suggesting a dynamic interaction between bodily self representation and social cognitive processes, here we aimed to investigate whether inducing a change in one’s own body representation may influence the space of interaction with other people. Specifically, due to the protective, safety value of IPS, we predicted that the experience of invisibility should induce participants to feel themselves more protected and less exposed during another person’s approach, thus leading to a significant contraction of IPS boundaries. To this aim, we measured IPS trough a comfort-distance task, in which participants were asked to stop an individual approaching them at a position in which they felt most comfortable with the other’s proximity^4, 5^.

In addition, to rule out the possibility that the invisible body illusion may simply influence space perception, we also assessed how individuals encode the reaching space near the body. In the neurocognitive domain, the reaching space has been conceptualized as a sensorimotor interface for the body to act on nearby objects. This working or reaching space^23^ has been termed peripersonal space (PPS), and it refers to the region of space coded in a body-centred reference frame by multisensory neurons^24^. In the present study, we measured PPS through a reaching-distance task^25, 26^ adapted to be similar to the methodology used to assess IPS, with the exception that, in this case, participants were asked to stop the other person at the distance in which they thought they could reach her.

Thus, IPS and PPS were measured using a similar methodology, through a comfort-distance and a reaching-distance task, respectively, which were repeated twice: before and after an invisible body illusion. Due to its effects on aspects of social cognition, we expected that the experience of having an invisible body should reduce the size of IPS, without affecting PPS extension.

On the contrary, if the illusion of invisibility modifies the perception of the space around the body per se, a modification of this space should be found independently from the social or sensorimotor valence of the task, and thus involving both IPS and PPS.

## Experiment 1

### Methods

#### Participants

Twenty four participants, all females, to avoid possible gender differences effects^27, 28^, volunteered for the study (age range = 20–26; mean age = 22.63). Sample size was determined a priori by conducting a power analysis using G*Power 3^29^. A small to medium effect size (${\eta }_{p}^{2}$ = 0.20) was specified based on a previous study conducted in our laboratory^30^. Within our chosen sample size and effect size, the power (1 − β) was approximately 0.80.

Participants were naive to the experimental hypothesis, and had no self-reported history of neurological or psychiatric disease. All participants had normal or correct to normal vision. They provided written informed consent to participate in the experiments, which were approved by the Ethical Committee of the University of Bologna, in agreement with the 2008 Helsinki Declaration. Participants were instructed to wear a pair of trousers and a t-shirt.

#### Setting

For the entire duration of the experiment, participants wore a set of head-mounted displays, HMDs, (TRIVISIO VRvision Prototyping GmbH, 800 × 600 resolution, equals 1.4 M pixels and full colour, 42° diagonal field of View). The spacing between HMD’s oculars was adjusted for each participant to fit their inter-pupillary distance (55–72 mm adjustable). HMDs were connected, through a PC, to a synchronized HD webcam colour (Logitech HD pro webcam C920, full HD 1080p) placed on a tripod adjusted at the same height of the participant’s head. Participants were asked to stand upright in a fixed position 40 cm to the left of the tripod. In this way, through the HMDs, participants viewed in real time the part of the room filmed by the webcam, as if their point of view was that of the camera.

#### Procedure

Experiment was conducted in the same rectangular room (7.5 × 6.5 m). The experimental session included two tasks: (i) a comfort-distance judgment to assess social interpersonal space (participants indicated the comfort distance between themselves and a confederate) and (ii) a reaching-distance judgment, designed to assess peripersonal space (participants indicated the reaching distance between themselves and a confederate).

Testing began with a participant standing in a fixed position, 40 cm to the left of the tripod and the confederate standing, facing the tripod from a 5 meters starting position. The confederate was always the one moving toward the camera, which corresponded to the participant’s first person perspective. Participants provided both comfort-distance judgments (“stop the confederate at the distance you feel comfortable with her”), and reachability-distance judgments (“stop the confederate at the distance you think you can reach her”). The distance was measured with a digital laser meter, as the distance between the confederate’s chest and a fix point on the tripod just below the camera (Agatel, model DM 100, error ±0.003 m). This procedure was repeated twice in separate blocks of five trials for each condition: before and after 2 minutes of visuotactile stimulation to induce the invisible body illusion. The order of tasks was counterbalanced across subjects.

**Figure 1:**
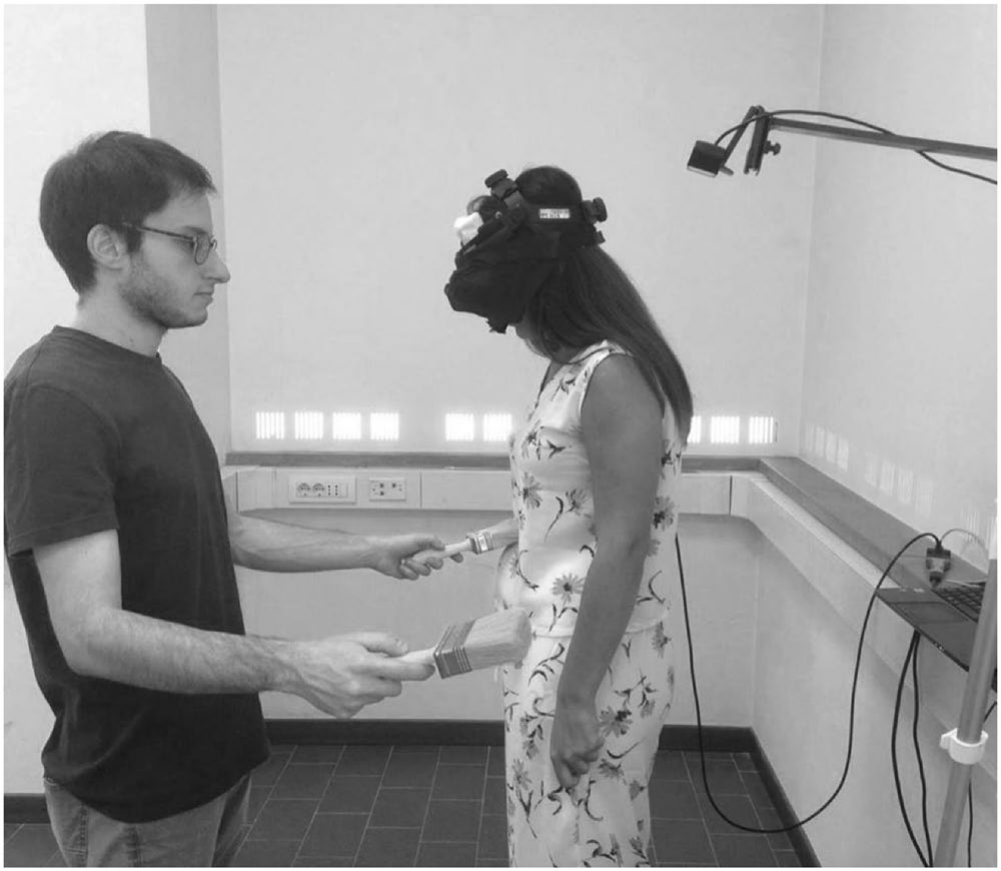
Experimental setup of the invisible body illusion. Participants watched in the HMDs the empty space captured by the camera. To induce the illusion, the experimenter applied touches to the participant’s body with a paintbrush and moved another paintbrush in the empty space in corresponding position.

The procedure to create the illusion of owning an invisible body was very similar to the one described in Guterstam *et al*.^22^. Participants were asked to close their eyes while the experimenter pointed the camera toward the floor. Then participants were asked to tilt their heads downwards as if looking at their bodies and open their eyes. In this way participants saw in the HMD the empty space captured by the camera where they expected to see their own body. To induce the illusion the experimenter stroked, with a large paintbrush, five different participant’s body parts while synchronously, in corresponding position, moving another paintbrush in the empty space under the camera (see Fig. 1; the experimenter (M.D.) shown in the figure gave written informed consent to publish his identifying image). According to the study by Guterstam *et al*.^22^, the strokes were delivered to the abdomen (A), the left and right lower arm (LLA, RLA) and the left and right lower legs and feet in a pre determined sequence: (A-A-A-A-LLA-LLA-LLA-LLA- RLA-RLA-RLARLA-LLF-LLF-LLF-LLF-A-A). This sequence was repeated two times. The duration of each stroke was 1 second and the interval between one touch and the next touch was 1.5 second. The entire visuotactile stimulation lasted about 2 min. To identify the portions of empty space corresponding to the stroked body targets of the invisible body, we used a female body as a template. Visual landmarks, which were out of participant’s view, indicated the starting and stopping points of brushstrokes. Since the work by Guterstam *et al*.^22^ has shown that the illusion of having an invisible body is dependent on spatio-temporal congruence of visual and tactile signals, as a control condition we applied asynchronous brushstrokes to the participant’s body and to the empty space, matching the total number and length of the strokes.

The experiment was conducted as a within-group counterbalanced design. Participants were randomly allocated to one of two groups, regarding whether they first received synchronous or asynchronous visuotactile stimulation, or vice versa. Synchronous and asynchronous stimulation were administered in two different sessions separate by one week.

Two different female confederates, unknown to the participants, were involved in the pre and post sessions. One of the confederates approached the camera for the entire duration of the first session before visuotactile stimulation, whereas the second confederate was introduced in the post experimental session. To avoid any aesthetical confound, the order of confederate facing the camera in the pre and post session was also counterbalanced between participants and within participant’s two conditions (Synchronous-Asynchronous visuotactile stimulation). Moreover, the two confederates were instructed to wear similar neutral clothes. During the approach toward the camera, the confederate walked with natural gaits at a constant speed. They were instructed to maintain a neutral expression and to keep their gaze looking straight ahead at a fixed point just below the camera.

To provide a measure of the illusory ownership of the invisible body, at the end of the experimental session, participants were asked to complete a 6-item questionnaire, which served to quantify the subjective experience of illusory ownership during multisensory stimulation. Questions were derived from Guterstam *et al*.^22^. Participants were asked to indicate the extent of their agreement or disagreement with six statements using a seven-point Likert scale, ranging from −3 (“I completely disagree”) to +3 (“I completely agree”), with a response of 0 indicating “neither agreed nor disagreed”. Three of the statements examined the perception of the illusion (S1–S3) and the other three statements were designed to control for suggestibility and task compliance (S4–S6) (see Table 1).

**Table 1:** Questionnaire statements.

During the experiment …
S1 I felt the touch of the brush in the empty space in the location where I saw the brush moving
S2 It felt as if I had an invisible body
S3 I experienced that the touch I felt was caused by the brush moving in the empty space
S 4 When I saw the brush moving, I experienced the touch on my back
S5 It felt as if I had two bodies
S6 I could no longer feel my body

Questionnaire used to evaluate the subjective experience after visuotactile stimulation: statements S1–S3 examined the perception of the illusion; statements S4–S6 were designed to control for suggestibility and task compliance.

## Results

**Figure 2:**
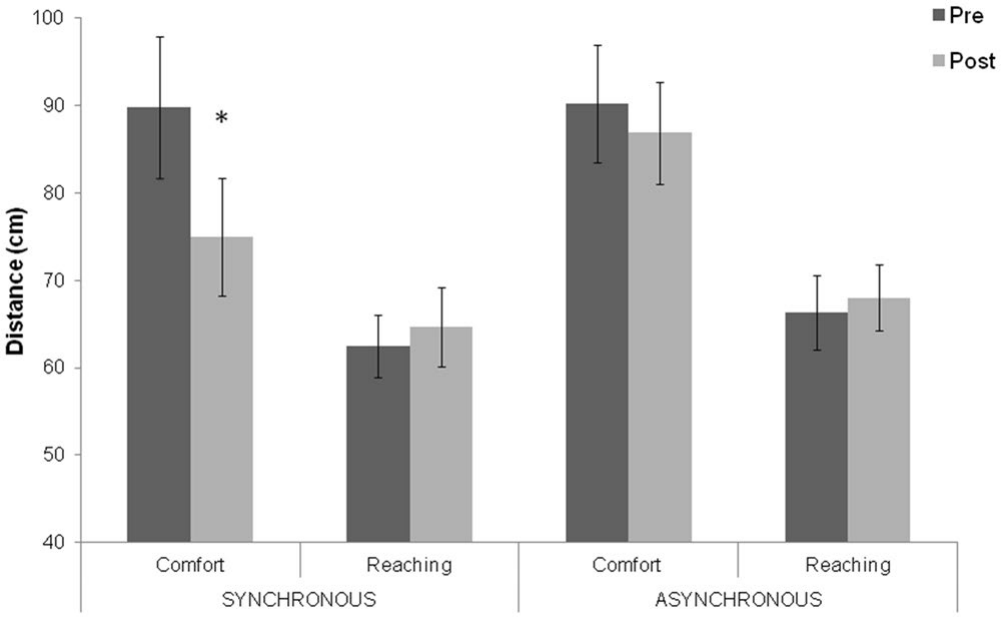
Effects of visuotactile stimulation on comfort and reaching-distance. Statistical comparison of mean distance (cm) in the two tasks (comfort and reaching judgment), in the stimulation conditions (synchronous and asynchronous visuotactile stimulation), and in the two sessions (pre- and post-visuotactile stimulation). Error bars indicate standard errors of the mean. The asterisk indicates a significant difference before and after invisible body illusion in the synchronous condition.

To test effect of the invisible body illusion on the comfort-distance and reaching-distance, the mean distances obtained in different experimental conditions were compared trough a three-way ANOVA, with Stimulation (synchronous vs asynchronous), Session (pre vs post visuotactile stimulation) and Task (reaching vs comfort-distance) as within-participants factors. Newman-Keuls post hoc test was used to analyze significant interactions. Data revealed a significant effect of the Task (F_1,23_ = 16.89; p < 0.0001; ${\eta }_{p}^{2}$ = 0.42) indicating that the participant-confederate distance was larger in the comfort than in reachability-distance task. The interaction Task × Session (F_1,23_ = 11.66; p < 0.01; ${\eta }_{p}^{2}$ = 0.33), as well as the interaction Stimulation × Session × Task were significant (F_1,23_ = 7.92; p < 0.01; ${\eta }_{p}^{2}$ = 0.25) (see Fig. 2). Specifically, our results show that comfort-distance was smaller after (75 cm) than before (89.8 cm) visuotactile stimulation in the synchronous condition (p < 0.0001), but not in the asynchronous condition (86.8 vs 90.21 cm, p = 0.28). Moreover, the comfort-distance after the synchronous visuotactile stimulation was significantly smaller than the comfort-distance measured before, as well as after asynchronous visuotactile stimulation (p < 0.0002, in both comparisons). Reachability-distance, instead, was not significantly different between pre and post visuotactile stimulation in both synchronous (62.4 vs. 64.6 cm, p = 0.10), and asynchronous conditions (66.3 vs. 68 cm, p = 0.43). In sum, the critical statistical interaction Stimulation × Session × Task indicates that only the synchronous, but not the asynchronous, visuotactile stimulation affected comfort-distance estimation. In contrast, neither synchronous nor asynchronous visuotactile stimulation modulated reaching-distance.

**Figure 3:**
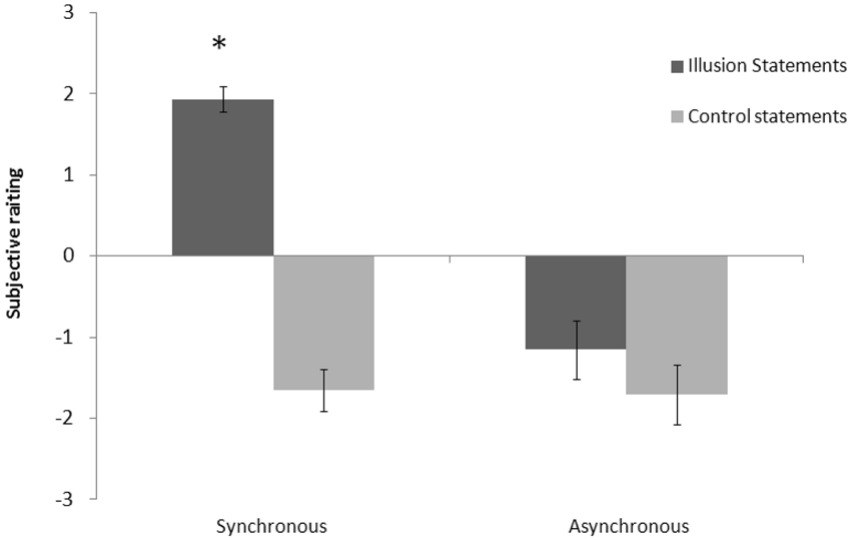
Questionnaire evidence for perceiving an invisible body. Mean score of illusion and control statements as a function of the condition (synchronous and asynchronous). Error bars indicate standard errors of the mean. The asterisk indicates a significant difference between illusion statements in Synchronous condition and all other conditions.

To investigate whether participants’ subjective experience during multisensory stimulation was affected by experimental conditions, the average rating of the illusion statements (S1–S3) and the control statements (S4–S6) at the questionnaire in the synchronous and synchronous conditions, were compared. An ANOVA with Stimulation (synchronous vs. synchronous) and Statement Type (illusion vs. control) as within-participants factors, showed a significant effect of Stimulation (F_1,23_ = 17.87; p < 0.00001; ${\eta }_{p}^{2}$ = 0.68), Statement Type (F_1,23_ = 102.78; p < 0.00001; ${\eta }_{p}^{2}$ = 0.66), and of their interaction (F_1,23_ = 61.22; p < 0.00001; ${\eta }_{p}^{2}$ = 0.72) (see Fig. 3). Participants in the synchronous stimulation affirmed more strongly illusion than control statements (p < 0.001), and more strongly than in the asynchronous stimulation (both in the illusion and control statements) (p < 0.001 for all comparisons).

These findings show that IPS, as measured by the comfort-distance task, considerably reduces after synchronous, but not asynchronous, visuotactile stimulation. Since, as assessed by the questionnaire, visuotactile stimulation evoked illusory ownership over an invisible body only when touches are synchronously applied, the reduction of IPS is due to the perception of one’s own body as invisible. By contrast there was no significant difference in the reaching space between pre and post visuotactile stimulation both in the synchronous and asynchronous stimulation. This suggests that perception of one’s own body as invisible does not affect the general perception of space around the body, but it has unique effect when this space assumes a safety value in the comfort-distance task.

An alternative explanation of this result can be that reduction of social interpersonal space is not caused by the invisible body illusion per se, but it is due to an altered feeling of presence in the spatial environment as filmed by the camera and presented to the participants through the HMDs^31^. In other words, it is possible that simply the perception of the other person by means of the HMD may have led participants to feel themselves more shielded, and less exposed to the other’s approach. Related to this issue, participants could have particularly emphasized that what they saw did not correspond to their real first person perspective or to the real position of their body, since they were located 40 cm to the left of the tripod.

Therefore, to rule out the possibility that comfort-distance reduction reflects a general bias due to the HMD’s device and virtual reality system, we conducted another study (Experiment 2) aimed to modulate PPS, without altering social IPS. That is, by using the same methodology used to assess IPS and PPS of the Experiment 1, we investigated the possibility to reveal the opposite dissociation between these spaces. To this aim, we implemented a tool-use paradigm, known to affect PPS^30, 32–34^, adapted to the virtual reality system used in the previous experiment. Accordingly, we hypothesized that the active use of a tool should enlarge PPS, but leaving IPS unaffected, thereby confirming that variation of IPS size found in Experiment 1 is not merely the consequence of being in a virtual environment.

## Experiment 2

### Methods

#### Participants

A new group of female participants (n = 24; age range = 20–28; mean age = 23.91), naive to the purpose of the study, participated in Experiment 2. All participants had normal or correct to normal vision, no self-reported history of neurological or psychiatric dieses and all but three were right-handed, as assessed by Edinburgh Handedness Inventory^35^. They provided written informed consent to participate in the experiments, which were approved by the Ethical Committee of the University of Bologna, in agreement with the 2008 Helsinki Declaration.

#### Procedure

**Figure 4:**
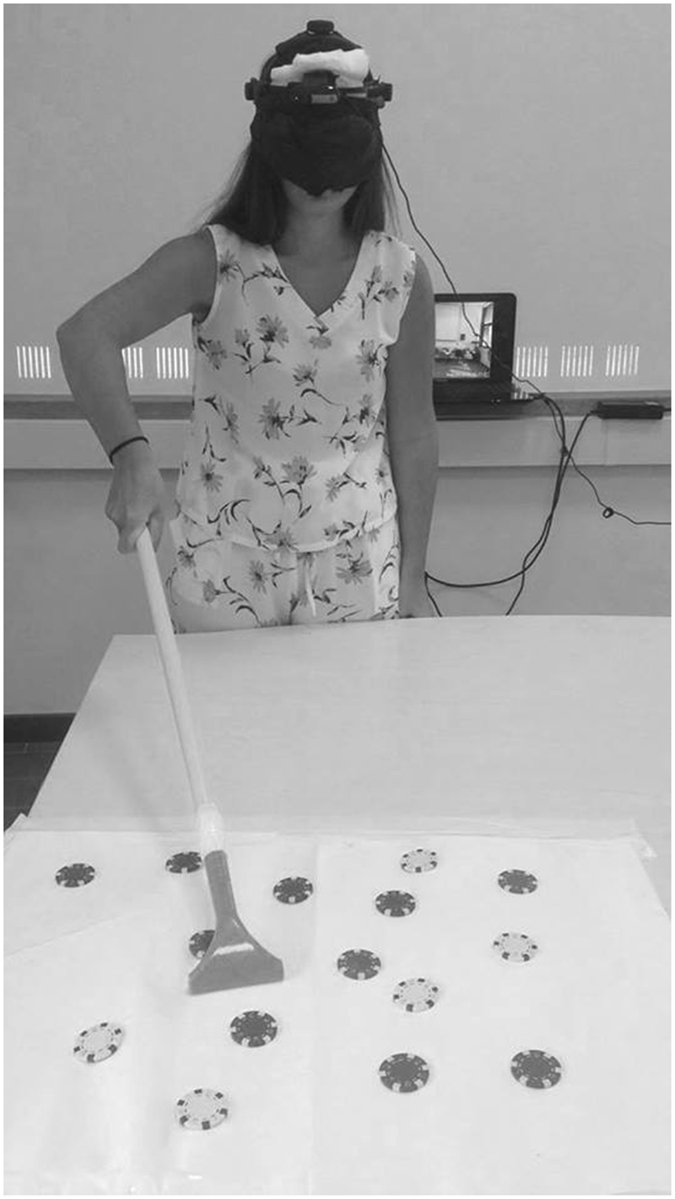
Experimental setup of tool-use. A webcam was applied on the HMDs worn by the participants. Participants were instructed to reach and retrieve different tokens.

In the Experiment 2, participants performed reachability and comfort-distance task before and after 12 minutes of active and passive tool training. Experimental setting and procedure were similar to the Experiment 1, with the exception that in Experiment 2 there were two web cameras: one webcam was on the tripod, and the other one was applied on the head mounted display (HMD) worn by participants. During tool training, the webcam on the tripod was turned off and the webcam applied on the HMD filmed the training, that participants watched online through the HMD. In the active tool training, participants were required to use a 70 cm long rake to perform different tasks with their right hand, with which they were instructed to reach and retrieve, one at the time, different tokens placed on a table-top (see Fig. 4) at a distance of ≈85 cm from the participants’ sternum. In the passive tool training, participants held the tool passively with their right hand while they were asked to verbally report some characteristics of the tokens put near the tip of the tool. The experiment was conducted as a within-group counterbalanced design. Participants were randomly allocated to one of two groups, regarding of whether they first performed active or passive tool training, or vice versa.

## Results

**Figure 5:**
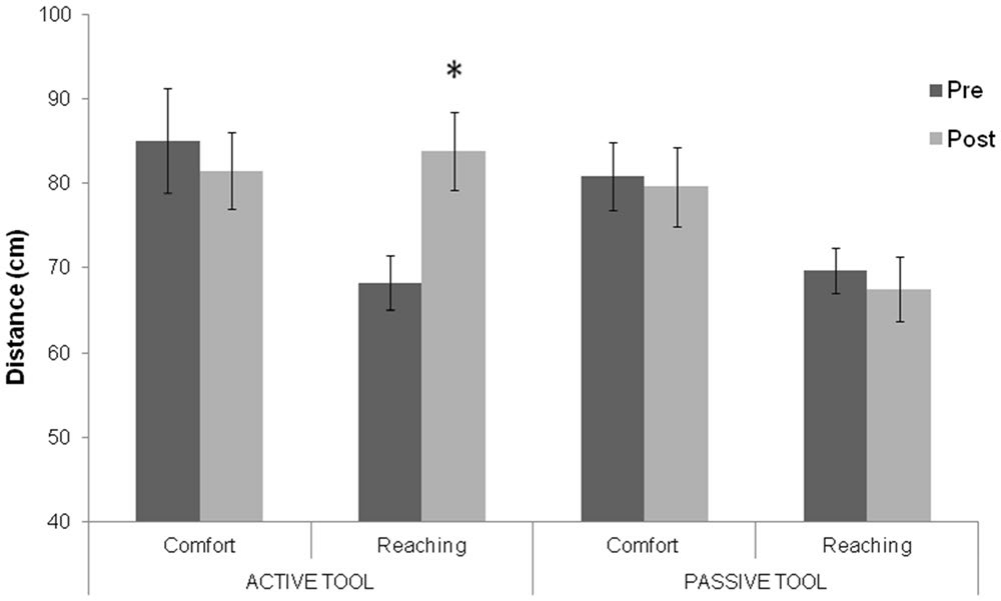
Effects of tool use training on comfort and reaching-distances. Statistical comparison of average distances (cm) in the two tasks (comfort and reaching judgment) in the two Tool training conditions (Active and Passive) and in the two Sessions (pre and post training). Error bars indicates standard errors of the mean. The asterisk indicates a significant difference in the reaching distance before and after active tool training.

To test the effect of the tool training on the comfort-distance and reaching-distance, the mean distances obtained in the different experimental conditions were compared through a three-way ANOVA with Training (active tool vs. passive tool training), Session (pre vs. Post tool training), Task (reaching vs. comfort-distance), as within-participant factors. Significant interactions were explored by Newman-Keuls post-hoc tests. The ANOVA showed a significant effect of the Task (F_1,23_ = 779.35; p < 0.05; ${\eta }_{p}^{2}$ = 0.16). As in Experiment 1, the participant-confederate distance was larger in the comfort-distance than in the reaching-distance task. Task × Session interaction (F_1,23_ = 15,46; p < 0.0001; ${\eta }_{p}^{2}$ = 0.40) as well as Training × Session × Task interaction were significant (F_1,23_ = 8.21; p < 0.001; ${\eta }_{p}^{2}$ = 0.26). Post-hoc tests revealed that the interaction was driven by an increased reaching-distance estimation after active tool use training (83.8 cm) with respect to before (68.2 cm, p < 0.01), whilst no significant difference between before and after active tool use was found in the comfort-distance task (85 vs. 81.5 cm, p = 0.57) In contrast, in the passive tool use training no significant differences before and after training were found in either reaching (69.7 vs. 67.4 cm; p = 0.80) or comfort-distance (80.8 vs. 79.6 cm; p = 0.72) (see Fig. 5). In sum, only active, but not passive, tool use training affected the reaching-distance estimation. Comfort-distance estimation was modulated neither by the active nor passive tool use training.

Experiment 2 shows an increased reaching-distance, but not change in comfort-distance, after an active tool use. No significant differences emerge in reaching and comfort-distance task after passive tool use. Thus, these findings are strongly in favor that the reduction of IPS found in Experiment 1 is not due to a low feeling of presence in the spatial environment presented in the HMDs because, in that case, we would found a reduction of IPS also in Experiment 2.

## Discussion

Available evidence suggests that IPS is modulated by higher order psychological and social factors concerning personality characteristics^2^, perception of social context^36^, and the attitude toward the interacting parts^5, 6, 9^. In the current study, in light of recent evidence emphasizing the importance of one’s own body representation in modulating interpersonal attitude^15–17, 20, 22^, we investigated whether a change in one’s own body representation can modify social IPS.

In Experiment 1, we show that the illusion of having an invisible body, elicited by temporally and spatially congruent visual and tactile stimuli (synchronous condition), and assessed by the questionnaire scores, significantly reduces IPS extension during the comfort-distance task. On the contrary, following the asynchronous control condition, the experience of the illusion was absent and, crucially, no significant modulation of interpersonal distance was found. These results therefore indicate that IPS reduction cannot be explained by effects that were non-specific to the illusion, such as, for instance, the mere habituation to the task. Rather, these findings support the close relationship between interpersonal distance and the bodily self-representation.

Despite synchronous visuotactile stimulation caused both a change in participant’s body perception, and a reduction of the space in which participants feel comfortable with the other’s proximity, it failed to modulate the participants’ judgement of reaching-distance. We found that reaching space (PPS) did not change either after synchronous or asynchronous stimulation. This latter result allows us to exclude that the observed reduction of IPS is merely due to a modification in the perception of space near the body after the invisible body illusion.

Nevertheless, one can argue that IPS reduction, rather than reflecting a change in participant’s body perception, is due to an altered feeling of presence in the spatial environment as observed through the HMDs. Perceiving other person’s approach by means of the HMD may have led participants to feel themselves less exposed and more protected as compared to a real, direct approach. This could be sufficient to induce a reduction of the space in which participants felt most comfortable with the confederate.

This interpretation of the findings, however, can be ruled out by the results of Experiment 2. In this latter experiment, a tool-use paradigm, known to modulate PPS^30, 32–34^, was adapted to the setting of Experiment 1. As predicted, after active tool-use, participants showed a significant enlargement of PPS, as assessed by the reaching-distance task, while social IPS remained unaffected. Thus, Experiment 2 reveals that viewing the surrounding environment through the HMDs does not hinder modulation of PPS by an appropriate manipulation (i.e., active tool-use). Crucially, the lack of IPS change in Experiment 2 suggests that the reduction of IPS observed in Experiment 1 cannot be accounted for by the feeling of protection associated with the virtual environment. Therefore, our overall findings clearly indicate that the reduction of IPS depends on the perception of one’s own body as invisible.

These results fit nicely with previous research by Guterstam *et al*.^22^, showing that invisible body illusion reduces the level of subjective stress and decreases heart rate in response to standing in front of a crowd of unknown people. Authors argued that if the body is represented as invisible, it will be represented as being invisible to outside observers as well, which in turn reduces social stress and anxiety response. Although in the current experiment we do not have a measure of subjective stress or level of anxiety during the confederate’s approach, this argumentation is particularly interesting for the present study. Indeed studies on IPS show that interpersonal distance is strongly modulated by alterations in brain areas involved in fear processing and anxiety responses, such as the amygdala^37^.

Thus, if participants truly experience invisibility, their body should be represented as invisible to others individual as well, which, in turn, might induce participants to feel more protected and less exposed during the confederate’s approach. As a consequence, participants may reduce the distance at which they feel more comfortable with the other’s proximity, allowing the confederate to be closer to their body. Related to this issue, an important finding of the present experiments is that interpersonal distance is consistently larger than reaching-distance, thereby indicating that participants feel comfortable when they cannot be reached and touched by an unfamiliar other. This finding is in line with an interpretation of IPS as a protective, safety space, while PPS represents a working space, or a space elected for reaching and manipulate close objects^23^.

Collectively, results of Experiment 1 and Experiment 2 provide converging evidence for a double dissociation between IPS and PPS. In Experiment 2, we found that the active use of a tool can temporarily alter the representation of the PPS, due to an extension of sensorimotor representation of arm length, as suggested by several previous studies^38–41^. Indeed, reaching space is modulated by, and relies on, morphological and sensorimotor body representation^38–43^. For instance, the size of near space is scaled as a proportion of one’s arm length^42^. This sensorimotor representation of the body morphology linked to PPS has been termed *body schema*, and is concerned with tracking and updating the positions and configuration of body parts in space^44, 45^. This representation typically does not enter into awareness, and is primarily used for spatial organization of action. Accordingly, in Experiment 1, no modification of PPS was found, since the invisible body illusion does not alter the sensorimotor representation used to guide action and act in space, i.e. the body schema.

In stark contrast, the invisible body illusion directly manipulates the conscious representation of the body external appearance, that is, the explicit body image^46–49^, as indicated by the questionnaire ratings. The term *body image* indeed refers to a distinct representation of the body used for perception of the body itself, primarily based on vision, but also on somatic perception, and represents the way the body appears to outside observers. It is not involved in action but plays a key role in emotional and social processing^50^. Therefore, the present findings not only reveal that IPS and PPS are two space representation functionally defined according to different behavioural context, but also suggest that IPS and PPS are linked to different high-order representations of the body, used for the perception (i.e., body image), and action (i.e., body schema) of the body, respectively.

Some may argue that the reduction of IPS found in the present research is not due to the feeling of body invisibility *per se*, but rather to a more general change in body form or appearance. Thus, in principle, any change in one’s own body perception might produce similar effects on IPS. Note, however, that Guterstam *et al*.^22^ have previously shown that the illusion of having a mannequin’s body did not induce the same feeling of protection and safety during a socially stressful situation. Thus, evidence from previous research makes it unlikely that reduction of IPS found in the current study was caused by a mere modification of the body appearance. However, we cannot exclude that other changes in one’s own body representation can modify the space of interaction with others. For instance, perceiving bodily similarity between oneself and others^15, 16^ may be another factor that could result in modulation of the IPS.

On the other hand, it is not our intent to claim that change of body image is the only way by which a modulation of IPS may occur. As mentioned above, IPS can be influenced by several psychological, social and context-dependent factors. Although in the current study the experience of having an invisible body, possibly through an increased sense of security^22^, reduces IPS, we do not exclude that feelings of safety and protection and a consequent reduction of IPS can be achieved through other manipulations unrelated to body image. For instance, interposing a transparent barrier between an observer and others may similarly cause the reduction of IPS without changing the observer’s body image.

Finally, the present findings indicating a close relationship between IPS and body representation may have significant implications for the study and treatment of different clinical disorders. For instance, recent studies have shown that children with autism spectrum disorder (ASD) have an altered IPS representation, preferring or larger^5, 6^ or closer^51, 52^ comfort-distances. People with social anxiety show an abnormal IPS too^8, 53^. Therefore, due to the close link between IPS and body image, we should expect an altered body image in ASD population or in people with social anxiety. Moreover, it should be interesting, as already suggest by Guterstam *et al*.^22^, to verify whether the effects of having an invisible body are stronger in people with social anxiety. Likewise, IPS is expected to be affected in populations with a persistent distorted body image, such as individuals with eating disorders^54^.
